# Pregnancy resumption following contraceptive discontinuation: Hazard survival analysis of the Indonesia Demographic and Health Survey Data 2007, 2012 and 2017

**DOI:** 10.1371/journal.pone.0264318

**Published:** 2022-02-23

**Authors:** Maria Gayatri, Budi Utomo, Meiwita Budiharsana, Gouranga Dasvarma

**Affiliations:** 1 Centre for Research and Development on Family Planning and Family Welfare, Badan Kependudukan dan Keluarga Berencana Nasional, East Jakarta, Indonesia; 2 Faculty of Public Health, University of Indonesia, Depok, Indonesia; 3 College of Humanities, Arts and Social Sciences, Flinders University, Adelaide, Australia; University of Salamanca, SPAIN

## Abstract

**Objective:**

The objective of this research is to estimate the probability of pregnancy resumption after discontinuing reversible contraceptives—pills, injectables, implants and IUDs, and to examine the factors associated with the resumption of fertility.

**Method:**

The study uses pregnancy calendar data from Indonesia Demographic and Health Surveys (IDHS) of 2007, 2012 and 2017. A hazard model survival method is used for estimating the time needed to resume pregnancy since discontinuing reversible contraceptives. Retrospective data on 4,573; 5,183 and 5,989 episodes of reversible contraceptive discontinuation at the three surveys respectively have been analysed.

**Results:**

This study shows that women regained fecundity within one year of discontinuing IUD, pill, injectables or implants. Women using IUD could resume their pregnancy faster than those using implants, pills and injectables. Over the three IDHS 2007, 2012 and 2017 the age-specific percentages of women becoming pregnant after one year of contraceptive discontinuation vary between 72 and 85 for IUD, 75 and 81 for pills, 72 and 76 for implants and 64 and 67 for injectables, with the percentages being higher among younger women. The analysis further shows that length of contraceptive use, parity, prior sexually transmitted infections, knowledge of fertile period, household wealth status and place of residence have no impact on occurrence of pregnancy after contraceptive discontinuation.

**Conclusion:**

The analysis disproves a myth that reversible contraceptives make women infertile. Depending on the type of reversible contraceptive used, 65% to 85% of the women were able to conceive after one year of discontinuation.

## Introduction

A reversible contraceptive is a method of contraception that makes it possible for a woman to conceive after use of the method has been discontinued [1]. In a review article, Mansour et al. show that a non-permanent or reversible contraceptive does not cause permanent infertility after the use of that contraceptive is discontinued [2]. The reversible contraceptives studied in this paper are oral contraceptives (pills), injectables, Intrauterine Devices (IUD) and implants, which have a high degree of efficiency to avoid pregnancy during use. Under perfect use conditions, the failure rate for pill, injectables, IUD and implants are less than 2 pregnancies per 100 women during the first year of use [3]. However, many women have a misconception that a long duration of use of these contraceptives may lead to infertility [4–7]. Such misconceptions have a negative impact on the utilisation of modern contraceptive methods in Indonesia and elsewhere [8–12]. For example, a study in Indonesia shows that the myths of oral contraception may dry the vaginal or can harm women’s uterus that leads a delay in conceiving [12]. Moreover, it is very rare for a woman to use long acting reversible contraception (LARC) before having at least one child. Data collected at the 2007, 2012 and 2017 Indonesia Demographic and Health Surveys (IDHS) show that not even one woman used LARC like IUD or implants before her first pregnancy.

Further, several studies have reported that women experience a delay in becoming pregnant after the discontinuation of LARCs [2, 13–17], as indicated by the low prevalence of pregnancy following the cessation of the use of contraceptives. Return of fertility after discontinuation of the LARC methods such as IUD, pills and implants appears to be faster than that after the discontinuation of injectables due to the women’s hormonal effects. Duration of contraceptive use, age, parity, women’s knowledge on reproductive health (especially of fertile period), occupation, smoking and alcohol consumption have been reported as factors influencing the return of fertility following contraceptive discontinuation [2, 4, 14, 15, 18, 19].

Indonesia has a success story in family planning program. The contraceptive prevalence rate increased sharply from 8% in the early 1970s to about 60% in 2002 [20]. In 2017, contraceptive prevalence rate in Indonesia was about 64%. However, the prevalence of Long-acting and Permanent methods in Indonesia is still low (13.4%) compared with injectables (29%) [21]. One year contraceptive discontinuation rate for all methods increased from 21% in 2002 to 29% in 2017 [21].

In Indonesia, many women do not know that their fertile period lasts for a short duration during a menstrual cycle. Knowledge about a woman’s fertile period is important for planning pregnancies, but only 22% of women of reproductive age are able to identify accurately when their fertile period is between two menstrual cycles [21]. Understanding the fertile period is essential for couples to improve their chance of getting pregnant. This information is also important for those women who stop their contraceptive methods for the reason of desire to become pregnant.

The main objective of the study is to estimate the probability of pregnancy resumption after the discontinuation of reversible contraceptives. For this purpose, pregnancy calendar data from the 2007, 2012 and 2017 rounds of Indonesia Demographic and Health Survey, have been analysed. This study also identifies the factors associated with the resumption of pregnancy after contraceptive discontinuation.

## Materials and methods

### Data collection

This study is based on household and women’s individual recoded datasets including the reproductive health calendar obtained from IDHS 2007, 2012 and 2017. The consistency of results obtained by analysing data from the three independent Indonesia Demographic and Health Surveys mentioned above strengthens the conclusions of the study. The study is restricted to retrospective follow-up of episodes beginning 62 months prior to each survey date and ending in the three months before each survey date.

The reproductive health calendar is a tool used for collecting detailed information on pregnancies, births, contraceptive use, discontinuation of contraceptive use and marital status for each month spanning the five years prior to each survey [22]. Calendar data records month-by-month reproductive calendar history and contraceptive events of each respondent for a period of five years preceding the survey [22]. The first column of calendar data records complete information on women’s reproduction such as births (written as “B”), pregnancies (written as “P”), terminations (written as “T”) and contraceptive use (categorized as “0” for no method, “1” for female sterilization, “2” for male sterilization, “3” IUD, “4” for injectables, “5” for implants, “6” for pill, “7” for condom, “8” for female condom, “9” for diaphragm, “K” for lactational amenorrhea, “L” for rhythm, and “M” for withdrawal). The second column of calendar data records information on the reasons for contraceptive discontinuation. The reasons are coded as “0” for infrequent sex/husband away, “1” for became pregnant while using, “2” for wanted to become pregnant, “3” for husband/partner disapproved, “4” for wanted more effective method, “5” for side effects/health concerns, “6” for lack of access, “7” for costs too much, “8” for inconvenient to use, “F” for up to God/fatalistic,”A” for difficult to get pregnant/menopausal, “D” for marital dissolution, and “X” for other. In this calendar data, women are asked “Why do you stop using the methods?” for each episode of contraceptive discontinuation.

This research is limited only to women who have discontinued their contraceptive methods because they wish to be pregnant, while other reasons for discontinuation are excluded from the analysis. The unit of analysis in this study is the episode of contraceptive use among reproductive age (15–49 years) women during 5 years before the survey.

The analysis is based on the last episode for women who have multiple episodes of contraceptive discontinuation. To analyse the calendar data, information containing a number of string functions is extracted with the help of a statistical software called Stata version 15.1 (Stata Corp, College Station, TX). Extraction of the calendar data enables the calculation of Century Month Code (CMC) to assess the length of calendar suitable for research purposes.

### Variables

We have used data based on the respondents’ own reports about their discontinuation of reversible contraceptives to become pregnant. The time elapsed between contraceptive discontinuation and the start of pregnancy is compared for each reversible contraceptive used before discontinuation. A single-decrement table is generated to calculate the episodes of time to pregnancy after contraceptive discontinuation, where the decrement starts at the point in a woman’s reproductive calendar when she discontinues the use of a reversible contraceptive to become pregnant. The decrement stops at the point of time when the woman reports becoming pregnant.

The analysis in this study has two control variables, namely the time from baseline (start of the particular contraceptive use by a woman) to onset (time of contraceptive discontinuation by the woman) and time from onset to the time of survey. The potential explanatory variables examined in this study are based on their relationship observed in previous studies, and include duration of use of the contraceptive [23, 24], woman’s age [4, 15, 17, 18], woman’s smoking status [18, 25], woman’s occupation (not working, blue-collar worker, white collar worker) [26], and woman’s place of residence (urban-rural). The descriptive statistics for women who discontinue reversible contraceptive methods are presented as frequencies and percentages (Table 1).

**Table 1:** Percentage distribution of women who discontinuing contraceptives due to desire to be pregnant according to the use of long-acting reversible contraceptives and various socio-demographic characteristics. Indonesia 2007, 2012 and 2017.

Contraceptive method/socio-demographic characteristics	IDHS 2007 (n = 4,573)	IDHS 2012 (n = 5,183)	IDHS 2017 (n = 5,989)
	Percentage	Percentage	Percentage
Long acting reversible contraceptive			
Injectables^*)^	60.1	63.7	64.9
Pill	30.6	30.0	27.4
IUD	4.2	3.4	3.8
Implant	5.1	2.9	3.9
Duration of use			
< 2 years	50.6	52.7	49.1
2–4 years	38.0	37.5	38.2
4 years and above	11.4	9.8	12.7
Age (years)			
15–24	16.7	14.9	12.9
25–34	59.2	60.9	58.4
35 and above	24.1	24.2	28.7
Frequency of sexual intercourse			
< Less than twice per week	60.6	60.2	62.0
≥Twice or more per week	39.4	39.8	38.0
Type of work			
Blue-collar	40.0	45.6	39.1
White-collar	9.1	9.6	12.0
Not working	50.9	44.8	48.9
Smoking status			
Yes	15.3	20.7	22.4
No	84.7	79.3	77.6
Place of residence			
Urban	41.4	45.2	44.1
Rural	58.6	54.8	55.9
Region			
Java Bali	60.6	59.8	60.1
Outer Java Bali	39.4	40.2	39.9

Note: n denotes the sample size.

Source: Calculated by Authors based on Indonesia Demographic and Health Survey 2007, 2012 and 2017 [21, 41, 42].

### Data analysis

The time to return to pregnancy post contraceptive discontinuation in the context of the return of fertility is measured by calculating the time (in months) taken from stopping a reversible contraceptive method to the beginning of pregnancy, as reported at each survey by the women. The calendar data contain information about the last month of contraceptive discontinuation for planning a pregnancy. The time to return to pregnancy is calculated by counting the number of months from the time of contraceptive discontinuation to the time the woman became pregnant. If the woman was not pregnant at the time of the survey, her data are regarded as censored. The results are presented in terms of the distribution of time to return of fertility (Table 2).

**Table 2:** Cumulative percentage distribution of women who discontinuing contraceptives due to desire to be pregnant by time to return to pregnancy following discontinuation of pills, injectables, IUD and Implants. Indonesia 2007, 2012 and 2017.

Survey	Contraceptives discontinued in order to conceive	Cumulative percentage of women conceiving at 3, 6, 12, 18 and 24 months following contraceptive discontinuation				
		3 months	6 months	12 months	18 months	24 months
IDHS 2007	Pill (n = 1,398)	53.6	67.9	80.7	89.5	91.8
	Injectables (n = 2,747)	35.4	49.2	67.1	78.8	83.7
	IUD (n = 193)	50.7	70.6	79.0	83.3	84.2
	Implant (n = 235)	42.5	58.5	75.7	80.7	82.1
IDHS 2012	Pill (n = 3,303)	50.7	64.2	78.0	84.2	87.4
	Injectables (n = 1,554)	31.1	46.0	64.7	77.3	82.4
	IUD (n = 175)	63.7	75.3	84.9	88.4	88.4
	Implant (n = 151)	45.8	53.1	71.9	81.2	84.6
IDHS 2017	Pill (n = 3,887)	42.6	59.3	75.4	81.4	86.0
	Injectables (n = 1,641)	27.7	42.9	64.2	76.6	83.5
	IUD (n = 228)	42.8	59.6	71.5	75.9	82.7
	Implant (n = 233)	46.7	59.1	74.9	82.8	85.7

Note: n denotes the number of women who discontinued the given contraceptive at each survey.

Source: Calculated by authors based on Indonesia Demographic and Health Survey 2007, 2012 and 2017 [21, 41, 42].

The time to pregnancy following the discontinuation of a reversible contraceptive method is a discrete variable, which shows the duration of time *‘t’* (in months). The main analysis of this study is survival analysis which is assumed to begin at the time of discontinuation of a reversible contraceptive method and end at the time of the occurrence of pregnancy. Right-censoring of episodes of stopping contraception for reasons of becoming pregnant occurs if the episode is still in progress (i.e. the woman has not yet become pregnant) at the time of the survey. Under such circumstances, the full duration of the episode (to pregnancy) remains unknown, but can be estimated by assuming that the censored cases have the same probability of occurrence of the event as non-censored cases. The outcome of time to pregnancy can be: "a successful outcome", indicated by the occurrence of pregnancy, or "a censored outcome", indicated by no occurrence of pregnancy at the time of survey. The population in this study comprises women of reproductive ages (15–49 years) who discontinue their reversible contraceptive methods for reasons of becoming pregnant.

The analysis of data in this study is done in two steps. The first step consists of descriptive statistics based on percentage distribution of women according to use of long-acting reversible contraceptives and various socio-demographic characteristics of the women from data collected at IDHS 2007, 2012 and 2017; and the time (in months) when the women returns to pregnancy after the discontinuation of each of the LARC methods, namely pill, injectables, IUD and implants. The second step provides estimates of survival probabilities (in this case the probabilities of becoming pregnant) after the discontinuation of a LARC. This estimation uses a product-limit formula of the Kaplan-Meier method [19].

In this study, as mentioned above the Kaplan-Meier procedure is used for estimating the probability of women who have conceived at the time *t*_*(f)*_ following the discontinuation of reversible contraceptives. The log rank test is employed to compare more than two survival groups based on the observed and expected cell counts [27]. Log rank test has been performed to compare the differences in the survival function between reversible contraceptive methods (pills, injectables, IUDs, and implants). The median time to pregnancy in survival analysis is calculated based on the number of months elapsed when 50% of women have become pregnant. The median is chosen as a measure of average in preference to the mean because the survival time to pregnancy is frequently highly skewed as some women become pregnant too soon or too late after discontinuing their use of a contraceptive. The analysis includes the estimation of cumulative probabilities that women become pregnant at 3, 6, 12, 18 and 24 months after stopping their reversible contraceptive methods.

Finally, multivariable Cox regression has been used for analysing significant predictors of pregnancy after the discontinuation of reversible contraceptives. A time-dependent Cox model has been used in this study because the model does not satisfy the proportional hazard assumption [27]. This model is known as extended Cox model and is calculated with time dependent predictors [27]. The assumption of proportional hazards has been tested graphically as well as by using a goodness-of-fit test.

All p-values used in this study are two-tailed. We used individual-level sampling weights to produce nationally representative results. Sampling weights have been applied to adjust for the unequal probability of selection between cases for certain areas [28]. The analyses are adjusted using the complex survey design which performs survival analysis. Complex sample design is mostly used for large national surveys in which the subjects have unequal probabilities of selection as respondents and characterized by probability weight, primary sampling unit (clustering /block census) and stratification [29]. Moreover, complex sample is needed to analyse survey data to minimize biased point of estimates which deal with clustering and stratification of the national survey data [29]. We conducted all analyses by using Stata IC 15.1 which can handle the survival analysis with complex survey design to incorporate the strata, cluster and probability of weights into the analysis by adding the effect of survey estimation [svy] in the Stata do-file.

### Ethics approval

Data of the 2007, 2012 and 2017 Indonesia Demographic and Health Survey did not attach any personal identity. However, the authors had received ethical approval from the Ethical Committee the Faculty of Public Health University of Indonesia. Permission to use IDHS data was obtained from The Demographic and Health Survey (DHS) Program.

## Results

Table 1 shows the distribution of women according to episodes of reversible contraceptive use and socio-demographic characteristics in a set time (in months) after contraceptive discontinuation to plan a pregnancy. More than 60% of the episodes of contraceptive use consisted of the use of injectables. Injectables are the most popular contraceptive method in Indonesia as reported at IDHS2007, 2012 and 2017. The episodes of the use of long acting reversible contraceptives like intra-uterine devices (IUD) and sub-dermal implants are much lower with only 3%-5% of the women using them in 2017.

About 60% of the episodes of contraceptive use discontinuation have occurred among women aged 25–34 years. Of all the episodes of contraceptive use discontinuation, about one half have occurred to women who used contraception for less than two years, less than 40% occurred to women who used contraception for two to four years, while about 10% of women used contraception for four years or more. More than three-quarters of women who discontinued the use of reversible contraceptives never smoked.

Table 2 shows the percentage distribution of women by time to return to pregnancy following discontinuation of reversible contraceptives to become pregnant. These distributions are shown for the years 2007, 2012 and 2017 IDHS respectively. A general observation from the table is that success in returning to pregnancy is higher among discontinuers of pill and IUD users and that more than 80% of the women discontinuing any of the four contraceptives have become pregnant after 24 months of discontinuation.

The table further shows the net cumulative pregnancy rates at 3, 6,12,18 and 24 months after discontinuation of reversible contraception for each of IDHS 2007, 2012 and 2017. The cumulative prevalence of reported pregnancies increases as the length of exposure to pregnancy after contraceptive discontinuation increases. At six months after contraceptive discontinuation, 50% or more of the women discontinuing contraception reported to have become pregnant. Over the three IDHS mentioned above, the cumulative percentage of women conceiving at six months after stopping the use of an LARC varies between 60–75 for IUD users and 53–59 for implant users. The corresponding percentages for short-term reversible contraception varies between 60–68 for pill and 43–49 for injectables. By one year after contraceptive discontinuation, very large percentages of women are reported to have conceived. These percentages, over the three IDHS 2007, 2012 and 2017 are 72–85 for IUD, 75–81 for pills, 64–67 for injectables, and 72–76 for implants. More than 82% of women stopping the use of reversible contraceptives have reported becoming pregnant at two years following contraceptive discontinuation.

Table 3 shows the median length of time to return to pregnancy following contraceptive discontinuation to plan a pregnancy. It is based on the number of months by which 50% of all women are reported to have become pregnant after discontinuing the use of each LARC. The median is found to be 2–4 months for IUD, 3–4 months for pills, 4–5 months for implants, and 7–8 months for injectables indicating that, compared to discontinuers of pill, IUD and implants, discontinuers of injectables take a little longer to conceive. The differences in the median length of time to become pregnant between pill users and injectable users, and between IUD users and injectable users are statistically significant.

**Table 3:** Median delay to pregnancy following contraceptive discontinuation due to desire to be pregnant.

Contraceptive Methods	Median	
	Median	95% CI
IUD	2–4 months	2–6 months
Oral contraceptives	3–4 months	3–5 months
Implants	4–5 months	3–7 months
Injectables	7–8 months	6–8 months

Source: Calculated by the authors based on Indonesia Demographic and Health Survey 2007, 2012 and 2017 [21, 41, 42].

Table 4 presents, for each of IDHS 2007, 2012 and 2017 the hazards ratios showing the relative influence of the type of reversible contraceptives discontinued to have children and socio-demographic factors on the length of time elapsed since discontinuation to become pregnant. Time dependent Cox regression has been used for estimating the outcome variable, which in this case is the length of time to fertility resumption after contraceptive discontinuation. Compared to the discontinuation of injectables, the time needed to conceive after discontinuation of pills and IUD is much shorter at all the three IDHS, and these differences in time are statistically highly significant. Even though the time needed to resume pregnancy after discontinuing implants is even shorter compared to that after discontinuing injectables at all the three surveys, these differences are statistically not significant. The association between duration of contraceptive use before discontinuation and the length of time to resume pregnancy is statistically not significant. In this study, women’s age, frequency of sexual intercourse, time elapsed since contraceptive discontinuation until interview, women’s occupation, women’s smoking status and the region of women’s residence have been used as control variables. Based on the results of hazard ratios, it can be seen that younger women become pregnant faster than older women. From another perspective, women who do not smoke (actively or passively) also become pregnant faster than women who smoke.

**Table 4:** Factors influencing fertility resumption after discontinuing the use of reversible contraceptives to conceive.

Independent Variable	IDHS 2007 (n = 4,573)			IDHS 2012 (n = 5,183)			IDHS 2017 (n = 5,989)		
	n	B	HR and (95% CI)	n	B	HR and (95% CI)	n	B	HR and (95% CI)
Methods									
Injectables*^)^	2,747		1	3,303		1	3,887		1
Pill	1,554	0.53	1.70 (1.51–1.91) **	1,554	0.55	1.74 (1.53–1.98) **	1,641	0.47	1.60 (1.44–1.77) **
IUD	193	0.56	1.59 (1.21–2.08) **	175	0.79	2.20 (1.64–2.95) **	228	0.36	1.44 (1.15–1.80) **
Implant	235	0.02	1.02 (0.77–1.34)	151	0.09	1.09 (0.83–1.42)	233	0.09	1.09 (0.88–1.36)
Duration of use	4,573	-0.01	0.99 (0.98–1.01)	5,183	-0.01	0.99 (0.99–1.01)	5,989	-0.01	0.99 (0.98–1.02)
Age	4,573	-0.01	0.99 (0.98–1.00) *	5,183	-0.02	0.98 (0.98–0.99) **	5,989	-0.01	0.99 (0.99–1.00) *
Frequency of sexual intercourse.									
< 2x/week	2,773		1	3,120		1	3,712		1
≥ 2x/week	1,800	0.03	1.03 (1.01–1.13) *	2,063	0.02	1.02 (1.01–1.12) *	2,277	0.09	1.11 (1.01–1.21) *
time*method	4,573	0.01	1.01 (1.01–1.02) **	5,183	0.01	1.02 (1.01–1.02) **	5,989	0.01	1.01 (1.01–1.02) **
time DO-interview***	4,573	0.01	1.01 (1.00–1.01) **	5,183	0.01	1.00 (1.00–1.01) **	5,989	0.01	1.00 (1.00–1.01) *
Occupation									
Blue-collar worker	1,831		1	2,361		1	2,343		1
White-collar worker	415	0.01	1,00 (0,82–1,22)	500	0.07	1.02 (0.78–1.09)	720	0.13	1.14 (1.02–1.29) *
Not working	2,327	0.14	1,15 (1,05–1,27) **	2,322	0.14	1.15 (1.05–1.26) **	2,926	0.23	1.26 (1.17–1.36) **
Smoking									
Yes	698		1	1,075		1	1,343		1
No	3,876	0.25	1,28 (1,10–1,37) **	4,108	0.02	1.12 (1.02–1.13) **	4,646	0.09	1.92 (1.85–1.99) *
Region									
Java Bali	2,772		1	3,100		1	3,598		1
Outer Java Bali	1,801	0.19	1,22 (1,12–1,33) **	20,83	0.14	1.15 (1.06–1.25) **	2,391	0.22	1.25 (1.16–1.34) **

Note

*sig. at α 0.05

**sig. at α 0.01

***(DO: *Drop Out*); CI = Confidence Interval; HR = Hazards Ratio; CI = Confidence Interval.

Source: Calculated by Authors based on Indonesia Demographic and Health Survey 2007, 2012 and 2017 [21, 41, 42].

## Discussion

Approximately about 85% of women reported becoming pregnant in the first year, which means 15% of women failed to become pregnant within one year [30]. The ability to become pregnant after discontinuing contraceptives, especially with the reason to become pregnant, is an essential concern for reproductive women. The time to return to fertility after contraceptive discontinuation depends on the type of contraceptive discontinued [31].

The analysis has revealed that the percentage of women becoming pregnant at one-year post contraceptive discontinuation are lower for women stopping the use of injectables than for women stopping the use of pills, implants, and IUDs. The median time to pregnancy is the fastest for women who discontinued the use of IUD, followed by the median time to pregnancy for women who discontinued the use of pills, implants and injectables respectively.

The above findings indicate that, among reversible contraceptive methods, the time to return to pregnancy for the non-hormonal methods is shorter than that for hormonal methods. This is in line with findings of studies which show that the prevalence of pregnancy at one year after contraceptive discontinuation ranges from 79.4% to 95% for oral contraceptive, 71.2% to 96.4% for IUD (Copper Intrauterine Devices and Levonorgestrel-releasing intrauterine system), and 37.5% to 85.6% for implants [2]. Based on a meta-analysis study, about 71%-96% (with an average of 84.75%) of women who removed their IUDs became pregnant in the first one year [31]. However, for injectables, the prevalence of pregnancy at one year after contraceptive discontinuation in the present study, namely 64%-67%, are lower than the prevalence of pregnancy reported in the literature, which is 72.5% to 82.9% [2]. The reason for this difference is probably under reporting of the termination of pregnancies after discontinuing the use of implants, especially terminations in the first trimester of pregnancy.

The effect of contraceptive discontinuation on subsequent fertility shows that more than 82% of women conceive within 24 months of its stopping based on three rounds data of IDHS. However, the median time to pregnancy is faster about 2–5 months for IUDs, pills and implants, and about 7–8 months for injectables. Based on the findings, there is no evidence in support of the perceived side effects of contraception on future infertility, and women do not need to worry about the effect of contraception on their future fertility once they have discontinued the use of the contraceptive in order to have a child. Contraceptive methods are beneficial in preventing unwanted or unplanned pregnancies. Moreover, there are some non-contraceptive benefits of contraception such as prevention of pelvic inflammatory diseases, rheumatoid arthritis, menstrual migraine, and a reduction of the risk of ovarian, cervical and colorectal cancer and ectopic pregnancy [32, 33].

This study has also shown that the cumulative probability of pregnancy for ex-contraceptive users depends on age. Younger women are found to resume pregnancy faster than older women, and this negative relationship between women’s age and time to pregnancy may be attributed to the quantity and quality of women’s eggs, both of which gradually decrease with increasing of age [34–36]. Other reasons could be a reduction in the frequency of sexual intercourse and an increase in the risk of spontaneous abortion with increasing age [37, 38].

Smoking status is found as one of the factors associated with the length of time needed to return to pregnancy. In this study, women who are not smokers are found to have a higher prevalence of pregnancy than those who are smokers, as indicated by a hazards ratio of 1.1 to 2.0 across the three surveys. This can be attributed to the fact that smoking is related to lower fecundity rates at each stage of reproduction [39]. Active and passive smoking are also associated with increased risk of infertility and menopause among women aged under 50 years [25]. However, it can not be ruled out that women who smoke might be older than women who do not smoke.

Duration of contraceptive use is found not to be associated with reduced time to conception following contraceptive discontinuation. It is also found that, in line with the findings of other studies, the duration of use of a contraceptive before its discontinuation to plan a pregnancy does not appear to be associated with the time to return to fertility [4, 14, 15, 18, 31, 40].

### Limitations of the study

This study has a few limitations. First of all, this analysis excludes all nulliparous women who had used any LARC method before their first pregnancy. This omission resulted in the inability to measure the conception rate for the first pregnancy after the discontinuation of methods like IUD or implant. This has also prevented any comparison of the time to return to pregnancy between nulliparous and multi-parous women. Lastly, detailed information on the first day of the last menstrual period of the women is not available in this study, and without this information, it is not possible to calculate the respondent’s exact time to become pregnant. Thus, the time to become pregnant, as the dependent variable has been calculated from the month women stopped using a contraceptive method to plan a pregnancy up to the month when the women reported they became pregnant. In other words, the calculation of the time to return to pregnancy may not be as accurate as desired. However, this is not considered to affect the findings in any significant manner.

## Conclusion

This study dispels the myth that use of reversible contraceptives causes permanent infertility. The findings suggest that previous use of contraceptive methods does not cause any impairment to the capacity to conceive after discontinuing the use of contraceptives. Against the Indonesian common beliefs, the findings defy that the use of any reversible contraceptives cause permanent infertility. Analysis of data from IDHS 2007, 2012 and 2017 show that the prevalence of pregnancy in the first year after discontinuation are found to be between 75% and 81% for contraceptive pills, between 72% and 76% for implants, between 72% and 85% for IUDs and between 65% and 67% for injectables. The prevalence of pregnancy after two years of discontinuation for all four contraceptives are found to be between 82% and 92%. Duration of contraceptive use before discontinuation is found to have no negative effect on the ability of women to conceive.

## Recommendations

Information about the near certainty of resumption of fertility or ability to conceive within a year of the discontinuation of reversible contraceptives should be disseminated widely through information, education and communication campaigns in family planning, especially among reproductive age couples. This would eradicate the age-old belief that women, who have ever used reversible contraceptives are unable to conceive even after they have stopped using them. This message should be delivered through high-quality, comprehensive and clear contraceptive counselling to encourage women to use reversible contraceptives with adequate information about their reversibility within one year of discontinuing their use.
